# PICKLE 2.0: A human protein-protein interaction meta-database employing data integration via genetic information ontology

**DOI:** 10.1371/journal.pone.0186039

**Published:** 2017-10-12

**Authors:** Aris Gioutlakis, Maria I. Klapa, Nicholas K. Moschonas

**Affiliations:** 1 Department of General Biology, School of Medicine, University of Patras, Patras, Greece; 2 Metabolic Engineering and Systems Biology Laboratory, Institute of Chemical Engineering Sciences, Foundation for Research and Technology-Hellas (FORTH/ICE-HT), Patras, Greece; Parc de Recerca Biomedica de Barcelona, SPAIN

## Abstract

It has been acknowledged that source databases recording experimentally supported human protein-protein interactions (PPIs) exhibit limited overlap. Thus, the reconstruction of a comprehensive PPI network requires appropriate integration of multiple heterogeneous primary datasets, presenting the PPIs at various genetic reference levels. Existing PPI meta-databases perform integration via normalization; namely, PPIs are merged after converted to a certain target level. Hence, the node set of the integrated network depends each time on the number and type of the combined datasets. Moreover, the irreversible *a priori* normalization process hinders the identification of normalization artifacts in the integrated network, which originate from the nonlinearity characterizing the genetic information flow. PICKLE (Protein InteraCtion KnowLedgebasE) 2.0 implements a new architecture for this recently introduced human PPI meta-database. Its main novel feature over the existing meta-databases is its approach to primary PPI dataset integration via genetic information ontology. Building upon the PICKLE principles of using the reviewed human complete proteome (RHCP) of UniProtKB/Swiss-Prot as the reference protein interactor set, and filtering out protein interactions with low probability of being direct based on the available evidence, PICKLE 2.0 first assembles the RHCP genetic information ontology network by connecting the corresponding genes, nucleotide sequences (mRNAs) and proteins (UniProt entries) and then integrates PPI datasets by superimposing them on the ontology network without any *a priori* transformations. Importantly, this process allows the resulting heterogeneous integrated network to be reversibly normalized to any level of genetic reference without loss of the original information, the latter being used for identification of normalization biases, and enables the appraisal of potential false positive interactions through PPI source database cross-checking. The PICKLE web-based interface (www.pickle.gr) allows for the simultaneous query of multiple entities and provides integrated human PPI networks at either the protein (UniProt) or the gene level, at three PPI filtering modes.

## Introduction

Proteins play a fundamental role in the catalysis and regulation of cellular processes. In doing so, in most cases, they do not act alone, but their functionality emerges through their interaction with other proteins. The complexity of this vast protein-protein interaction (PPI) network, called the protein interactome, we are only beginning to understand, especially in human [1–4]. Thousands of small-scale or high-throughput experiments have been conducted to-date, each one revealing parts of the human interactome. Numerous public and commercial source PPI databases have been developed, tasked with the objective of collecting experimental PPI data from the literature, forming extensive primary PPI datasets [5–8]. However, it has been shown that source PPI databases display limited overlap in their datasets due to different objectives, curation rules and subsets of the literature that they process [9,10]. Hence, at the present time, a vast insight into the currently known interactome can only be achieved by means of integration of -primary PPI datasets. To this end, various PPI meta-databases have been created over the past few years [11–19]. The integration process is cumbersome due to PPI annotation discrepancies between the source databases, stemming from distinct curation rules, the use of incompatible interactor descriptors and the selection of different primary protein identifier types (i.e. gene, nucleotide sequence (mRNA), or protein (UniProt) IDs) with which PPIs are recorded. An effort to overcome this situation and move towards the establishment of consensus in regards to curation protocols has been carried out by the International Molecular Exchange (IMEx) consortium [9, 20]. Typically, these heterogeneous PPI networks are integrated via normalization; interactions are first converted to a certain target level of genetic reference and then merged. This top-down process, however, carries two drawbacks. Firstly, the node set of the integrated network is not standardized, but depends each time on the number and type of the combined sources. As a consequence, different meta-databases are not directly comparable, thus limiting our capability to evaluate the way in which the hitherto reconstructed human protein interactome expands over time. This lack of standardization may also lead to artifacts in the integrated PPI network originating from the currently unresolved part of the human proteome; the associated redundancy issues are an example arising when distinct reviewed and unreviewed UniProt IDs refer to the same protein entity. The second drawback is the irreversible nature of this integration process. The *a priori* normalization approach suspends the connection between the primary and the integrated PPI networks, thus hindering the identification of normalization artifacts introduced to the integrated network due to the inherent nonlinearity of the genetic information flow. The same gene may produce different protein isoforms, e.g. due to alternative splicing; or a single protein may be encoded by multiple genes as a result of gene duplications, e.g. the human α1- and α2-globin gene pair or the various histone gene families. Thus, the primary and the normalized PPI networks are not in one-to-one correspondence (non-isomorphic).

We, recently, introduced PICKLE (Protein InteraCtion KnowLedgebasE), a meta-database for the direct PPI network in human [10] and proposed a bottom-up solution to the node set standardization problem. Instead of relying on the source PPI datasets to dictate the node set of the integrated network, we adopted the use of the reviewed human complete proteome (RHCP) of UniProtKB/Swiss-Prot [21] as a standardized reference node set. Moreover, focusing primarily on direct physical PPIs, we introduced a PPI filtering protocol, according to which, only PPIs with at least one supporting experiment capable of suggesting direct interactions are selected from the source PPI databases. However, the issues associated with the irreversible nature of normalization remained unresolved. To this end, we designed and developed PICKLE 2.0, presented here, that introduces the concept of ontological integration as an alternative to the traditional integration via normalization, providing a major advance in the field of primary PPI dataset integration. PICKLE 2.0 relies on the reconstruction of the RHCP genetic information ontology network, which facilitates the integration of primary PPI datasets into a nonhomogeneous network without the need of any *a priori* transformations. Specifically, we employ the semantic equivalence between gene, nucleotide sequence (mRNA) and protein (UniProt) entities based on the structure of the genetic information flow. Firstly, we assemble the RHCP genetic information ontology network by connecting the corresponding genes, nucleotide sequences (mRNA) and proteins (UniProt entries). Integration is achieved by superimposing each primary PPI dataset on the RHCP ontology network linking the interactor identifiers of each PPI exactly as they are stored in the source databases, regardless of the level of genetic reference to which they refer. Thus, the ontological integration allows the integrated network to be reversibly normalized to any level of genetic reference without loss of source information for any PPI, establishing thus a direct correspondence between the integrated network instances at different levels of genetic reference and enabling primary PPI dataset cross-checking.

## Methods

### Sources of genetic information and protein-protein interactions

The PICKLE 2.0 genetic information ontology network is based primarily on the UniProtKB repository in conjunction with data from the GenBank (Gene and Nucleotide/RefSeq), Ensembl and ENA *Homo sapiens* assemblies. The PICKLE 2.0 PPI network is based on the integration of the (i) HPRD binary dataset, using the file ‘HPRD_ID_MAPPINGS’ to correspond each hprd ID to one nucleotide sequence (mRNA) ID (either RefSeq ID or EMBL ID) [5]; (ii) the BioGRID PSI-MI tab-delimited file for human using information from both the mitab and tab2 versions [6, 22]; (iii) the DIP PSI-MI tab delimited file for human [8]; (iv) the IntAct- and (v) MINT- designated datasets from the MIntAct [7] PSI-MI tab-delimited file, after removing the entries for which at least one interactor is non-human, i.e. the taxon ID (TaxID) is not 9606. The HPRD PPI dataset is the only source that we use that does not comply with PSI-MI standards. To overcome this limitation, we automatically convert any terms we encounter to their PSI-MI equivalent. Details on the reconstruction of the genetic information ontology and PPI networks of PICKLE 2.0 are provided in the Implementation section, below.

### PICKLE 2.0 architecture

PICKLE 2.0 utilizes a 2012 Microsoft SQL Server backend and relies on two different modules to operate. The first module is a data processing component, the role of which is the automated collection and assembly of data. The second module serves as a front end that provides a mechanism of executing meaningful queries. The data processing unit was written in C# and dynamic SQL was preferred over stored procedures to achieve high maintainability and testability. The front end was written in ASP.NET/C#; due to the high complexity of the functionality that it has to perform, it relies on stored procedures to operate. PICKLE 2.0 is fully automated and will feature regular updates after its initial release. We plan to update the genetic information ontology network every three months and the PPI network semi-annually. Past releases will always be available at the PICKLE website.

### PICKLE 2.0 web-based interface

PICKLE can be queried through an online interface, freely available at www.pickle.gr. For the visualization of the retrieved PPIs, the Cytoscape web plugin is used [23]. More information on querying PICKLE through the web interface is provided in the Implementation section below. An introductory video tutorial is also available at the website. In the ‘Downloads’ tab, the user may find (a) the current genetic information ontology network of PICKLE 2.0 in OWL format, (b) the current release of UniProt- and gene- normalized PPI networks of PICKLE 2.0 at all filtering modes in tabular.txt format and (c) previous releases, including the UniProt-normalized network of PICKLE 1.0.

## Results and discussion

### PICKLE 2.0 implementation

#### PPIs as relationships between polymorphic entities

**Fig 1:**
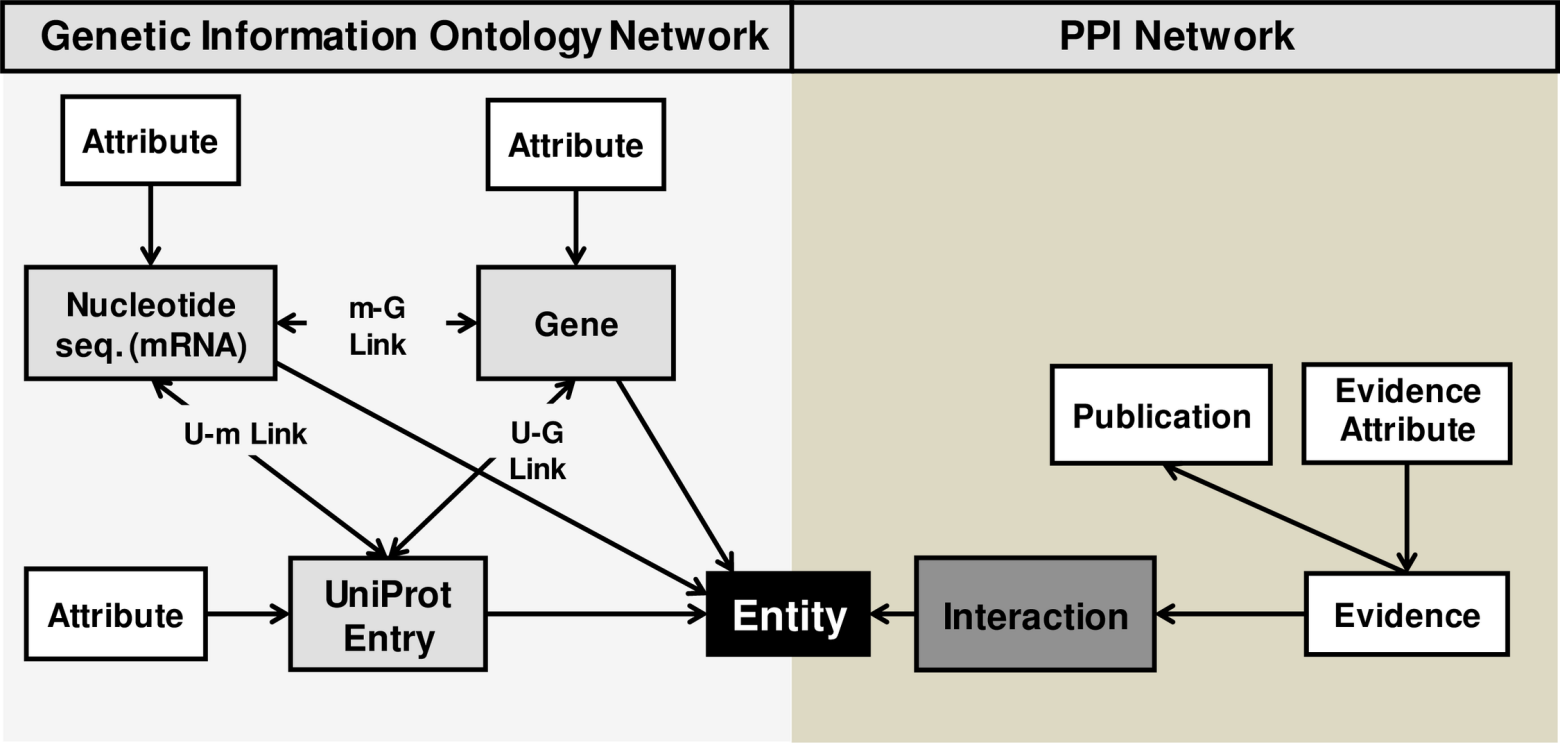
The underlying PICKLE 2.0 structure. The genetic information ontology network (left) comprises three classes of biological entities, UniProt Entry (U), Gene (G) and nucleotide sequence (mRNA) (m), linked through the genetic information flow. Primary PPIs are stored as pairings of biological entities forming a heterogeneous integrated network (right). Each stored PPI is associated with sets of evidence attributes. It should be noted that each set of evidence attributes is associated with a single or multiple publications.

In PICKLE 2.0, we define protein-protein interactions as links between *polymorphic entities*, instead of describing them as *polymorphic relationships* between classes of entities. This means that rather than accounting for all possible pairings of interactor identifier types encountered in the various source databases, interactions are defined as pairings of abstract entities at different levels of genetic reference, i.e. genes, nucleotide sequences (mRNA), or proteins (UniProt entries), described by various identifier types. These entities are interconnected through the genetic information flow and their relationships form the PICKLE genetic information ontology network (**Fig 1**). Having formed the latter, PICKLE replaces the typical means of primary PPI dataset integration through normalization with *ontological* integration. PPIs mined from source databases can now be superimposed on the genetic information ontology network and stored in their original form (**Fig 1**), without having to undergo any conversion to an *a priori* set level. In this way, we allow PPI dataset integration to precede normalization. An optimal view of the integrated PPI network can then be acquired at any level of genetic reference by reversibly traversing the ontology network, while the source information for each PPI is retained in its original form.

#### Reconstruction of the PICKLE genetic information ontology network

**Fig 2:**
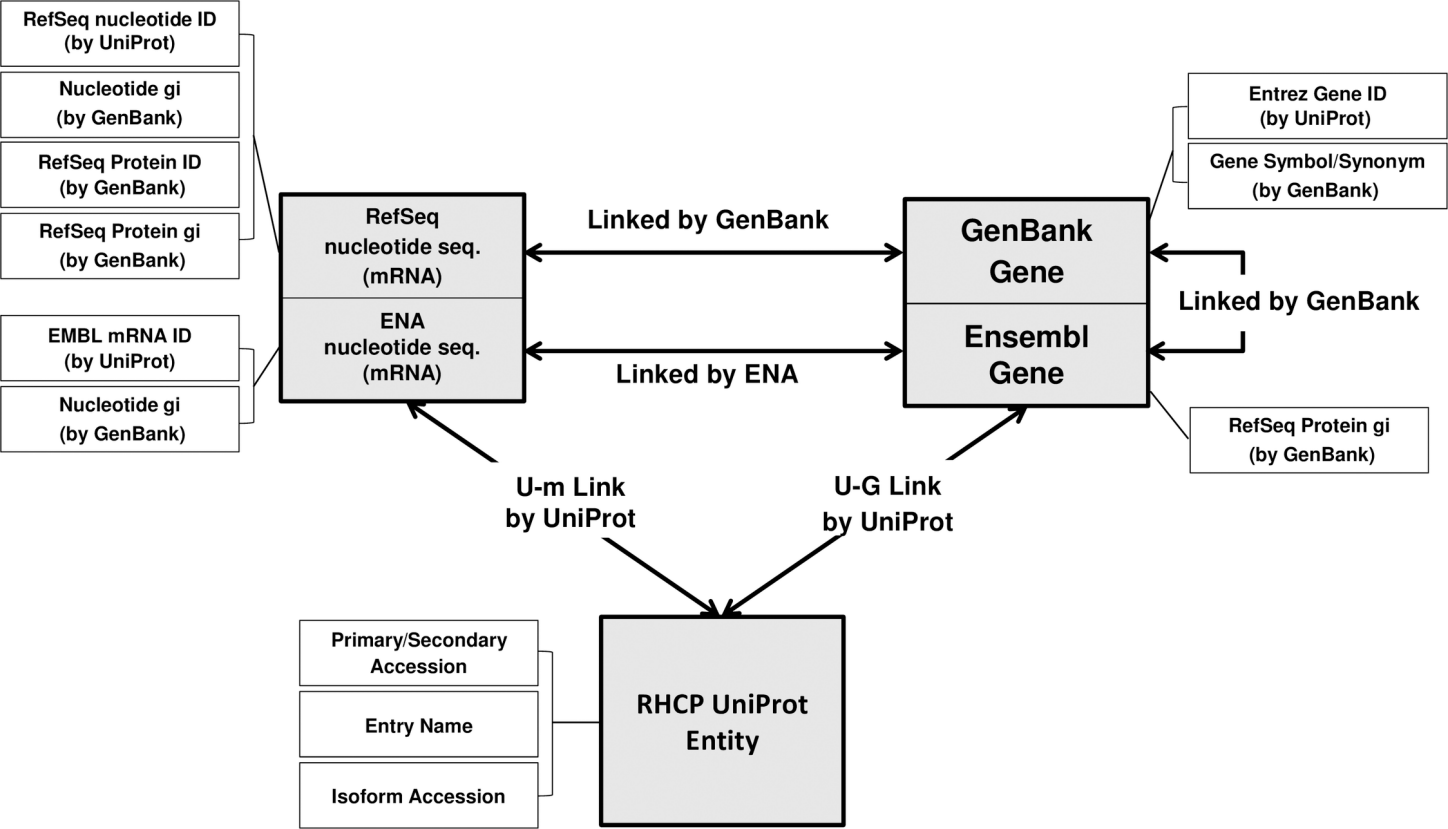
Data sources and entity identifier types for the reconstruction of the genetic information ontology network. The biological entity classes (gray boxes) are respectively populated with the RHCP UniProt entries and the RHCP-associated Entrez Gene IDs, Ensembl gene IDs, RefSeq and EMBL nucleotide IDs, according to UniProt. Other types of entity identifiers (all shown in white boxes) are also collected by a multitude of sources (shown in parentheses). The sources for the links between the various biological entities are shown, indicating the path of identifier normalization to the UniProt or gene level.

UniProt entries, genes and nucleotide sequences (mRNAs) are the three classes of biological entities constituting the PICKLE 2.0 genetic information ontology network (**Fig 1**). The “gene” class incorporates two sub-classes, comprising, respectively, entities from GenBank (defined by their Entrez Gene ID) and Ensembl (defined by their Ensembl gene ID) databases (**Fig 2**). Similarly, the “nucleotide sequence (mRNA)” class consists of two sub-classes, comprising, respectively, entities from Genbank (defined by their RefSeq nucleotide ID) and ENA/EMBL (defined by their EMBL nucleotide ID) databases (**Fig 2**). Based on the PICKLE principle of using the UniProtKB/Swiss-Prot RHCP as the reference protein interactor set [10], the reconstruction of the genetic information ontology network is initiated by populating the “UniProt Entity” class solely with the RHCP UniProt entries. Subsequently, the “gene” and “nucleotide sequence (mRNA)” classes are populated by Entrez Gene IDs, Ensembl gene IDs, RefSeq nucleotide IDs and EMBL nucleotide IDs explicitly associated with the RHCP UniProt entries, based on the UniProt cross-referencing with the respective databases (**Fig 2**). A set of attributes and other identifiers associated with each biological entity in the three classes and their sub-classes is also collected and stored using a multitude of sources (**Fig 2, S1 Table**).

The links between the entries in the “UniProt Entity” class and those in the “GenBank gene”, “Ensembl gene”, “RefSeq nucleotide sequence (mRNA)” and “EMBL nucleotide sequence (mRNA)” sub-classes are formed using the UniProt provided cross-references. When possible, Ensembl gene entries are associated to GenBank gene entries and EMBL to RefSeq nucleotide sequence (mRNA) entries, using information provided by GenBank. In this way, certain UniProt IDs that are associated only to Ensembl gene ID(s) by UniProtKB may indirectly become linked to Entrez Gene ID(s). The ontological interconnections between GenBank gene and RefSeq nucleotide sequence (mRNA) entries are retrieved from GenBank, while those between Ensembl gene and EMBL nucleotide sequence (mRNA) entries from ENA (**Fig 2**). Thus, the PICKLE 2.0 ontological network enables the association of an EMBL nucleotide sequence (mRNA) ID to Entrez Gene ID(s) through its association with Ensembl gene ID(s) retrieved from ENA; sequentially, the Ensembl gene ID(s) are linked to Entrez Gene IDs through GenBank. A typical consequence of database cross-referencing is the occasional retrieval of deprecated or obsolete entities. PICKLE 2.0 is equipped with an automated system that tracks and rectifies these discrepancies, by updating the deprecated and disregarding the obsolete entities. Any retrieved deprecated identifiers are stored as attributes of the active ontological entry, as shown in **S1 Table**.

#### Reconstruction of the PICKLE protein-protein interaction network

A. The “hybrid” PPI network. The primary datasets mined from the source databases that are used for the reconstruction of the PICKLE PPI network include the experimentally supported associations solely between biological entities in the PICKLE RHCP genetic information ontology network from (i) the HPRD binary dataset, represented as associations between nucleotide sequences (mRNAs), through the unique hprd ID–nucleotide sequence (mRNA) ID (either RefSeq or EMBL ID) mapping of HPRD, ii) the IntAct- and (iii) the MINT- designated datasets from MIntAct, (iv) the BioGRID, and (v) the DIP datasets, with both interactors designated with TaxID 9606. All genetic interference interactions (either in terms of interaction type or supporting detection method) in MIntAct, BioGRID or DIP are discarded. Other types of interactions, between protein and gene or nucleotide sequence (mRNA) entities, reported in MIntAct are still stored in PICKLE along with their supporting experimental evidence(s), but outside the typical PPI network, for the explicit purpose of primary PPI dataset cross-checking. Similarly treated are associations reported in BioGRID, which are supported by the experimental systems Protein-RNA or Affinity Capture RNA. After the initial data-mining process, the resulting datasets are combined into forming the hybrid *unfiltered* PPI network of PICKLE 2.0. Specifically, the retrieved interactions are recorded as links between the corresponding entity-nodes of the ontology network, without having to be normalized to a pre-set level of genetic reference. In this way, PICKLE 2.0 retains the original representations of each PPI in the source databases along with selected sets of evidence attributes (**S2 Table**), each set tied to its supporting publication(s) (**Fig 1**).

In PICKLE, what we call ‘evidence’ is an evaluated set of attributes provided by a source database regarding a specific PPI. In practice, the information extracted from each line in a PPI source file is analyzed and stored as a distinct evidence (**S2 Table**). Such a set of evidence attributes—is evaluated as “first-class” if suggesting a direct interaction. This category includes all evidence sets reported in BioGRID, MInAct, DIP, in which the interaction type indicates a direct interaction (**S3 Table**). In these cases, we trust the respective designation of the database curators, regardless of the rest of the supporting experimental data, i.e. the experimental system and/or detection method and the throughput (in the case of BioGRID and DIP) or expansion method (in the case of MIntAct). Even in the case of other interaction types, a set of evidence attributes is still evaluated as “first-class”, if the description of the supporting experimental setup accounts for methodologies capable of detecting direct interactions with a high degree of confidence (e.g. a yeast two-hybrid system). The list of experimental systems and detection methods included in this category for all source databases are shown in **S3 Table**. The rest of the evidence sets are designated as “second-class”, unless they are describing high-throughput experiments (in BioGRID and DIP) or depicting that the supported PPIs have been derived from spoke expansions (in MIntAct). In the latter cases, the described interactions would be considered of low probability of being direct and the evidence sets are designated as “third-class” (**S3 Table**). In summary, the “first-class” evidence sets are considered as strongly suggesting direct interactions. The “second-class” evidence sets may suggest direct interactions, but are subject to questioning and can be downgraded to a lower class designation given contrary information from other databases, as described below. The earlier described evidences referring to types of interactions between proteins and genes or nucleotide sequences (mRNAs) are given a “fifth-class” designation. In short, the integration of all primary PPI datasets results in a hybrid network comprising interactions between nodes of different entity types, supported by evidences of various designations. The unfiltered hybrid network of PICKLE comprises all interactions of the primary PPI datasets that are supported by at least “third-class” evidences.

B. PPI network normalization and standard filtering. The reconstruction of the final PICKLE PPI network requires the normalization of each interaction of the hybrid interactome to a set level of genetic reference. In PICKLE 2.0, this process can be arbitrary but it specifically refers to either the UniProt or the gene level. To transform a primary interaction to its normalized counterpart, we traverse the ontology network for each participating node towards the desired level (**Fig 2**). It is underlined that primary and normalized interactions remain linked, so no information loss occurs from this process and all normalization transformations are reversible. Each normalized interaction in the integrated network may be based on multiple primary interactions and aggregates the sets of evidence attributes recorded for all of them.

The sets of evidence attributes associated with primary PPIs assist in the definition and implementation of a standard filtering protocol for PPIs that have a low probability of being direct. As we are interested in reconstructing the direct protein-protein interactome in human, filtering PPIs based on their probability of being direct is of great value, as it can exclude a significant number of experimental uncertainties from the integrated network. In PICKLE 1.0, this protocol was applied before a primary PPI was normalized and integrated in the meta-database, thus non approved PPIs were rejected *a priori* and the filtering procedure was irreversible. In PICKLE 2.0, all primary PPIs mined from source databases are stored in their original form, superimposed on the genetic information ontology network; thus, both the filtered and the unfiltered integrated PPI networks, normalized at a selected level of genetic reference, can be accessed. The interactions in the integrated PICKLE network can be partitioned into thresholds of confidence of being direct based on the quality class of their supporting evidence. If a PPI is supported by at least one “first-class” evidence, it has a high probability of being direct. Thus, it is placed in the reciprocal, “first” confidence threshold and assigned a confidence score ‘1’. In a similar fashion, if a PPI is supported by at least one “second-class” evidence set, it has at least a moderate probability of being direct; thus, it is assigned to the second threshold with a confidence score ‘2’. The third threshold (confidence score ‘3’) contains all the interactions of low probability of being direct. All the above PPIs are included in the unfiltered PICKLE PPI network. PPIs supported only by quinary-class evidence sets, assigned a confidence score ‘5’, are not included in the unfiltered PICKLE PPI network, but are kept exclusively for cross-checking purposes. Excluding PPIs belonging to the third confidence threshold constitutes the standard PICKLE filtering mode and leads to the formation of the *standard* network in PICKLE.

C. PPI network refinement through primary PPI dataset cross-checking. A normalized interaction may be associated with multiple sets of evidence attributes, some of which corresponding to the same publication but reported by different source databases, each of which follows its own annotation and curation guidelines. Employing ontological PPI integration, PICKLE 2.0 enables the cross-checking between these primary PPI datasets in the cases of curation overlaps, leading to further refinement of the standard network from potential experimental artifacts. In all cases, “first-class” evidences retain their classification despite any potential discord between sources. However, “second-class” evidences provided from a particular database can be downgraded in the presence of alternative, lower-class (i.e. third or fifth) evaluations of the evidences provided from other sources for the same publication. Through this process, we assign two quality evaluations to each evidence: its original (standard) and its cross-checked class. Accordingly, each interaction is given two confidence scores; the cross-checked confidence score of a PPI is based on the cross-checked quality class of its supporting evidences. The second cross-checked threshold of PPI confidence (i.e. PPIs with cross-checked confidence score ‘2’, supported by evidences of at least second cross-checked quality class) comprises the PICKLE *default* PPI network.

**Fig 3:**
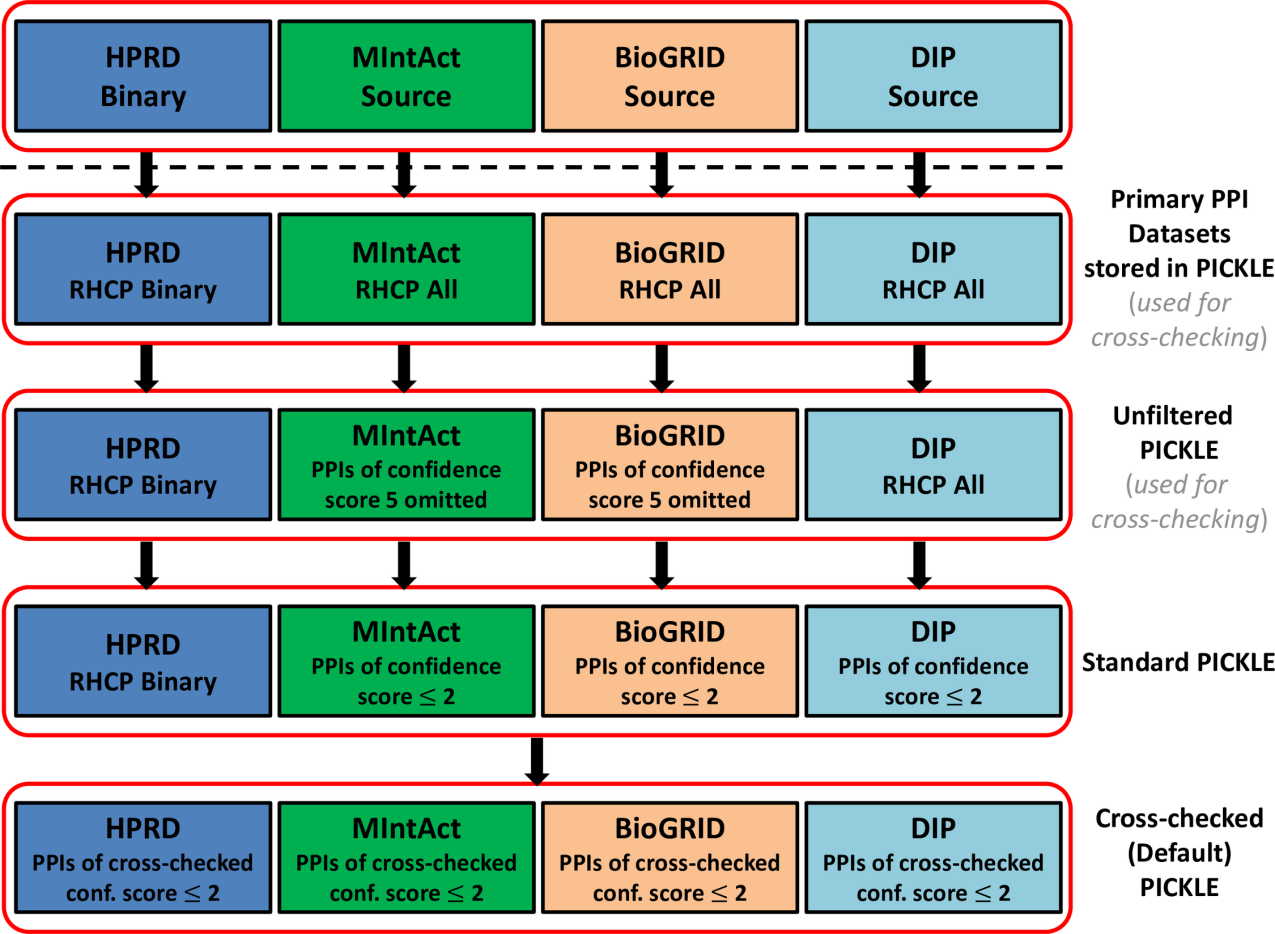
The HPRD, MintAct, BioGRID and MINT datasets involved in the unfiltered, standard and cross-checked (default) PICKLE 2.0 PPI network. RHCP stands for the reviewed human complete proteome. The primary datasets stored in PICKLE involve solely associations supported by at least one experimental evidence set with linked publication(s), with both interactors designated as human and belonging to the PICKLE RHCP genetic information ontology network. A confidence score ‘5’ suggests interactions of proteins with gene or nucleotide sequence (mRNA) entities.

Since interactions inherit their evidences from the hybrid network, situations arise where multiple normalized interactions are supported by the same evidence (i.e. an evidence describing an interaction at the gene level may be used to support multiple interactions at the UniProt level, if the participating genes encode multiple proteins). As a result of this, in many cases, our cross-checking process has an area of effect even across different normalization levels. It may end up taking into account and thus affecting multiple evidences across multiple interrelated interactions. In the exact same spirit, in certain cases, through the cross-examination of evidences referring to such interrelated interactions provided from different sources based on same publications, we may be able to designate certain normalized interactions as potential normalization artifacts. As the cross-checking filtering process enables the use of all available evidence from multiple sources to better evaluate the confidence score of a PPI being direct, the cross-checked PICKLE network is considered as the most refined from potential experimental and normalization artifacts. This is the reason that it is selected as the PICKLE default. Collectively, **Fig 3** shows the primary PPI datasets comprising the unfiltered, standard, and cross-checked (default) PICKLE 2.0 PPI networks.

#### Querying PICKLE via a web-based interface

**Fig 4:**
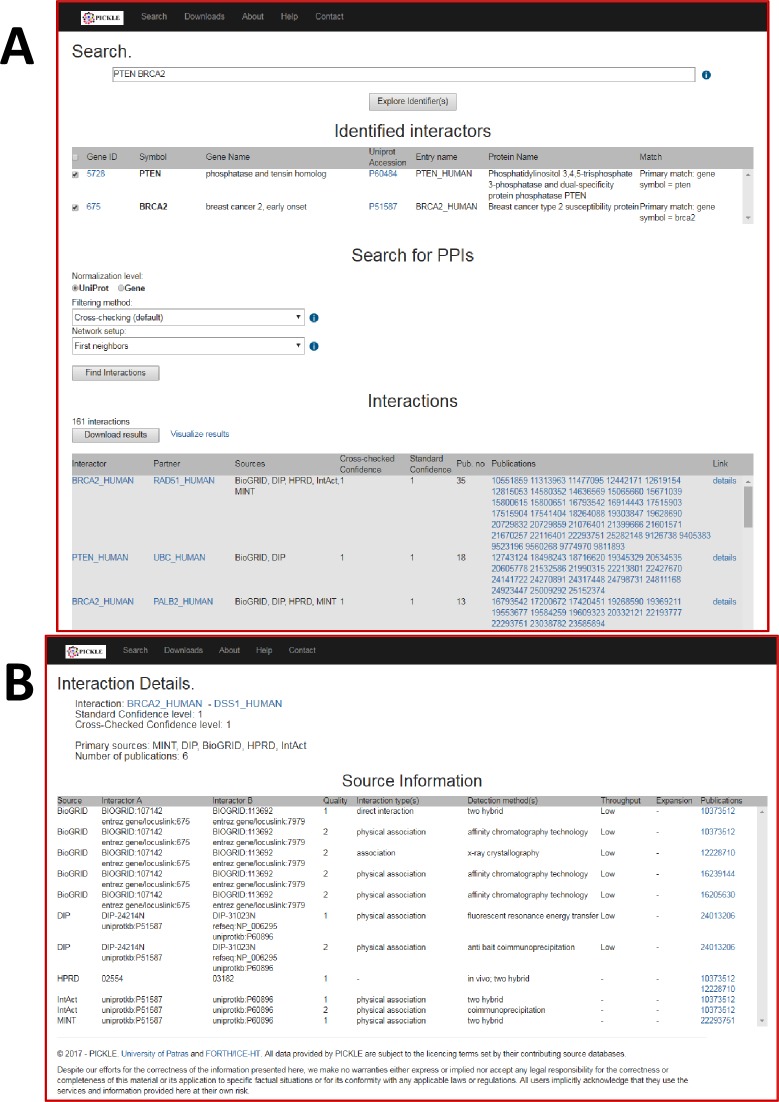
Snapshots of the PICKLE 2.0 web interface. (A) The entity identification and the interaction querying section followed by a part of the result section; (B) An example of the page that provides the original forms in which a PPI is reported by the source databases and the associated experimental evidences.

The online querying interface of PICKLE 2.0 (www.pickle.gr) comprises three parts: i. an entity identification section; ii. an interaction querying and result section, and iii. a network visualization section (**Fig 4**). Initially, the user enters one or more protein identifiers (almost any identifier type can be used, from UniProt, nucleotide sequence (mRNA) and gene accessions or names to GO terms and chromosomal regions). The system maps those identifiers to entities of the PICKLE RHCP genetic information ontology network and returns the candidate list to the user for review. Having selected at least one entity from the list, the user can proceed and set the PPI search criteria, namely a) the PICKLE network normalization level (i.e. UniProt or Gene), b) the PICKLE network filtering mode, i.e. none, standard or cross-checking (default), and c) the PPIs which the system will retrieve from the respective PICKLE network. Currently, the user can search for (i) the interactions of the queried biological entities, retrieving thus their “first neighbors”, or (ii) the interactions of the queried entities and any interactions that may exist between those first neighbors, or (iii) the interactions, if any, exclusively between the queried entities. The resulting interacting pairs are formatted in such a way that the first displayed interactor is always a queried entity. The interactions are sorted based on their cross-checked confidence score of a PPI, their standard confidence score and the total number of supporting publications, the links to which are also provided. The user can download the resulting PPI set in a tab-delimited format and/or visualize it (if size permits) via the Cytoscape web visualization plugin [23]. The user can also retrieve the source information about the identified PPIs as this is recorded in the primary datasets.

### The advantages of the PICKLE 2.0 structural scheme

The use of the UniProtKB-defined RHCP as the basis of the instantiation of the PICKLE ontological network offers a standardized point of reference between the PPI networks of successive versions and any future releases of PICKLE. In this way, by comparing the default network of PICKLE at the UniProt level between the two versions and in subsequent releases, we can directly and reliably evaluate the manner of expansion of the RHCP PPI network (and its contributing primary datasets). Moreover, with its structural scheme, based on the formation of the RHCP genetic information ontology network and the combination of the various primary PPI datasets through it without any prior transformation, PICKLE 2.0 achieves a reversible normalization process and a direct correspondence between the various normalized instances of the integrated PPI network. Hence, one can compare a PPI in the integrated network with all the different ways in which it was originally represented in the primary PPI datasets. This feature enables the identification of any potential normalization artifacts and a PPI reliability assessment through cross-checking of the available experimental evidence sets contributed by the various primary datasets. In addition, PICKLE 2.0 enables the storage of interactions between protein and gene or nucleotide sequence (mRNA) entities, e.g. those derived from protein-RNA or chromatin immunoprecipitation arrays/assays, as reported by certain sources, which can be used for purposes of cross-evaluating the supporting evidences provided in other primary datasets. In the same context, it is possible for assorted types of data (e.g. disease-related genes, genomic, transcriptomic or proteomic data) to be consistently integrated, viewed and interpreted in the context of the protein interaction network. In the next sections, we discuss these advantages through the analysis of the network of the first release of PICKLE 2.0 at the UniProt and gene levels and its comparison with that of PICKLE 1.0.

#### Evaluating the manner of expansion of the RHCP PPI network

The results of the comparison between the default PICKLE 2.0 network at the UniProt level with that of PICKLE 1.0 are shown in **S1 File**. The PICKLE 2.0 default interactome covers an additional 11.4% of the RHCP (**Table A in S1 File**) and shows a 59.1% increase in PPIs with respect to PICKLE 1.0. However, the number of common references between the three largest primary datasets (BioGRID, IntAct and HPRD) in PICKLE 2.0 remains very small (**Table B in S1 File**), justifying the need for multiple primary dataset integration for the reconstruction of a comprehensive PPI network in human. Notably, it concerns only 556 out of the 35752 references (~1.5%) supporting the PICKLE 2.0 (release 1) default network. This corresponds to an increase of just ~135, when PICKLE 2.0 contains 9,000 more references than PICKLE 1.0 (**Table F in S1 File**).

The observed increase trend in the number of UniProt IDs and PPIs between the two PICKLE versions supports our previous statement while analyzing PICKLE 1.0, that new experiments and incorporation of new references in the source databases are expected to reveal additional interactions concerning mostly the proteins already participating in the interactome, while most of the newly added UniProt IDs will have fewer than four interactions [10]. Indeed, comparison of the degree distribution between the two PICKLE versions (**S5 Table**) indicates that this is the case for most (83%) of the newly added UniProt IDs. Furthermore, most (63%) of the newly added interactions increase the number of UniProt IDs with degree larger than 10 and smaller than 100 and the number of UniProt IDs with more than 300 PPIs has more than doubled, i.e. 37 compared to 16 in PICKLE 1.0 (**S1 File, S5 Table**). A notable increase in PPIs is that of amyloid beta A4 protein (P05067) with 2010 interactions in PICKLE2.0 compared to only 124 in PICKLE 1.0, due to a targeted experiment [24] reported in BioGRID. The larger number of hubs in PICKLE 2.0 is in agreement with its network analysis results (**Table E in S1 File**), which indicate a higher degree of network connectivity compared to PICKLE 1.0. This is realized through: (i) a smaller diameter and number of isolated components, (ii) more shortest paths, and (iii) a larger clustering coefficient, network centralization and average number of neighbors.

#### Identifying sources of normalization biases by comparing the PICKLE 2.0 networks at the UniProt and gene levels

In PICKLE 2.0, we can holistically compare and observe the differences between the reconstructed human protein interactome at the UniProt and gene levels, because its structural scheme achieves a direct correspondence between the various normalized instances of the integrated PPI network at different levels of genetic reference. By comparing the default PICKLE 2.0 network between the UniProt and gene levels and moving from the unfiltered to the cross-checked (default) mode, we can assess the effect of normalization and/or experimental ambiguities on the topology of the reconstructed protein interactome. Such comparison and the respective results have not, to-date, been possible as the existing PPI meta-databases reconstruct the integrated network at a selected level of genetic reference after having normalized the primary datasets. All results of the comparison between the UniProt- and gene-normalized networks in PICKLE 2.0 can be found in **S2 File**; the main observations are presented here.

Globally, there is no substantial difference in the size of the human protein interactome between the two levels of genetic reference, because 97% of the interactors at a normalization level have one-to-one correspondence with their correspondents in the integrated network at the antipodal level. Thus, 97.3% of the PPIs at the UniProt level and 96.1% at the gene level have a single “sister” interaction at the other level. Despite this high apparent similarity, the two networks are not isomorphic and cannot be considered interchangeably. They have critical differences that need to be taken into consideration when collecting and using data from both instances. Specifically:

1. A part of the UniProt-normalized human protein interactome has no correspondent in the gene-normalized network. This comprises the PPIs with at least one interactor belonging to the RHCP UniProt entries, which UniProtKB has not yet corresponded to any Entrez Gene ID and this association could not be indirectly inferred in PICKLE by connecting the reported Ensembl ID to Entrez Gene ID based on GenBank (**Fig 2**).
2. The respective parts of the two networks involving UniProt or gene entities with multiple associations at the antipodal level have a much different topology. If these entities are of central location in the network and/or with a large number of first neighbors, the effect of the nonlinearity of the genetic information flow may not be confined to specific areas of the network but expanded to larger sections, thus modifying the corresponding network characteristics.

In PICKLE 2.0, our comparison indicated 106 interactors of the UniProt-normalized network, with up to 37 first neighbors, belonging to the RHCP UniProt entries with no correspondent Entrez Gene ID. In addition, more than 170 UniProt IDs are associated with multiple (up to 14) Entrez Gene IDs and 84 Entrez Gene IDs with multiple (up to 7) UniProt IDs. The full list of these entries and the number of their PPIs is provided in **S6 Table**. Most members of the histone protein family are associated with multiple genes, while members of the human leukocyte antigen (HLA) complex are an example of genes encoding multiple proteins. Taking into consideration that the group of UniProt IDs with multiple Entrez Gene IDs includes sixteen (18) with more than 50 (and up to 334) interactions, corresponding to at least two Entrez Gene IDs (in fact, histones H3.1 and H4 correspond to 10 and 14 Entrez Gene IDs, respectively), it becomes apparent that the corresponding part of the interactome at the gene level will be much denser than at the UniProt level, affecting other network characteristics as well. Similarly, the normalization to the UniProt level of the Entrez Gene IDs associated with multiple UniProt IDs may introduce invalid interactions. This issue could substantially modify the topology of certain parts of the UniProt-normalized network, if one considers that 18 of those Entrez Gene IDs with at least two associated UniProt IDs have more than 20 (and up to 270) interactions at the gene level. This type of concerns may become more prominent as BioGRID, the currently fastest growing source database reporting PPIs as interactions between Entrez Gene IDs, expands its contribution to the integrated interactome. Notably, BioGRID currently contributes 75% of the PPIs in PICKLE 2.0, being the unique source for 43.5% of them (**Table B in S1 File**).

To indicate the issues that arise from the nonlinearities in the genetic information ontology in the integration of multiple primary datasets referring to different levels of genetic reference, we use as an example the interaction between the Fascin actin-bundling protein 1 (gene symbol: *FSCN1*, Entrez Gene ID: 6624, UniProt ID: Q16658) and the GNAS complex locus protein (gene symbol: *GNAS*, Entrez Gene ID: 2778, UniProt IDs: O95467, P63092, P84996, Q5JWF2), which in PICKLE is mined by BioGRID and IntAct. BioGRID uses the Entrez Gene ID 2778 to identify the *GNAS* complex locus, while IntAct does so by specifically using the P63092 UniProt ID. When interpreting this interaction at the UniProt level, the description of IntAct is very specific about the PPI to which it refers, while this is not the case with BioGRID. The single interaction described as *GNAS*-*FSCN1* in BioGRID (at the gene level) explodes into four interactions (O95467-Q16658, P63092-Q16658, P84996-Q16658 and Q5JWF2-Q16658) at the UniProt level. In these cases, there is a high probability of introducing invalid interactions into the integrated network due to normalization artifacts. PICKLE 2.0 retaining the relationship between the normalized instance of a PPI with its original form(s) as provided from the source databases, enables the identification of these potentially invalid interactions, which can be taken into consideration in the interpretation of the acquired results. The ability of PICKLE 2.0 to directly correspond all normalized instances of the network at any level of genetic reference is expected to prove even more valuable as the resolution of the PPI network increases to the level of protein isoforms.

#### Identifying potential false positive PPIs in the PICKLE 2.0 network through primary PPI dataset cross-checking

PICKLE 2.0 provides a method for appraising normalization ambiguities by the combined examination of all primary datasets and the quality of the information that supports their data, producing the most refined “cross-checked” (default) PICKLE PPI dataset. For example, in the case of the previously mentioned interaction between the Fascin actin-bundling protein 1 and the GNAS complex locus protein, BioGRID indicates a physical association detected by a high throughput, affinity capture-mass spectrometry (MS) experiment, while IntAct a non-spoke physical association detected using anti bait coimmunoprecipitation. Based on the PICKLE standard evaluation scheme for the quality of evidences reported in BioGRID and MIntAct (see **S3 Table**), the BioGRID evidence is graded as “third-class”, while that of IntAct as “second-class”. In this way, while the unfiltered PICKLE 2.0 network at the UniProt level comprises all four PPIs derived from the normalization of the BioGRID-provided interaction, in the standard PICKLE network only the IntAct-designated Q16658-P63092 remains. While this process is a step in the right direction as it allows the detection and filtering of potential false positive interactions with a high degree of certainty, it is limited by the fact that the evidence provided for each PPI is evaluated independently by the various sources. The structure of PICKLE 2.0 enables further filtering of the PPIs through evidence set cross-checking between sources.

**Fig 5:**
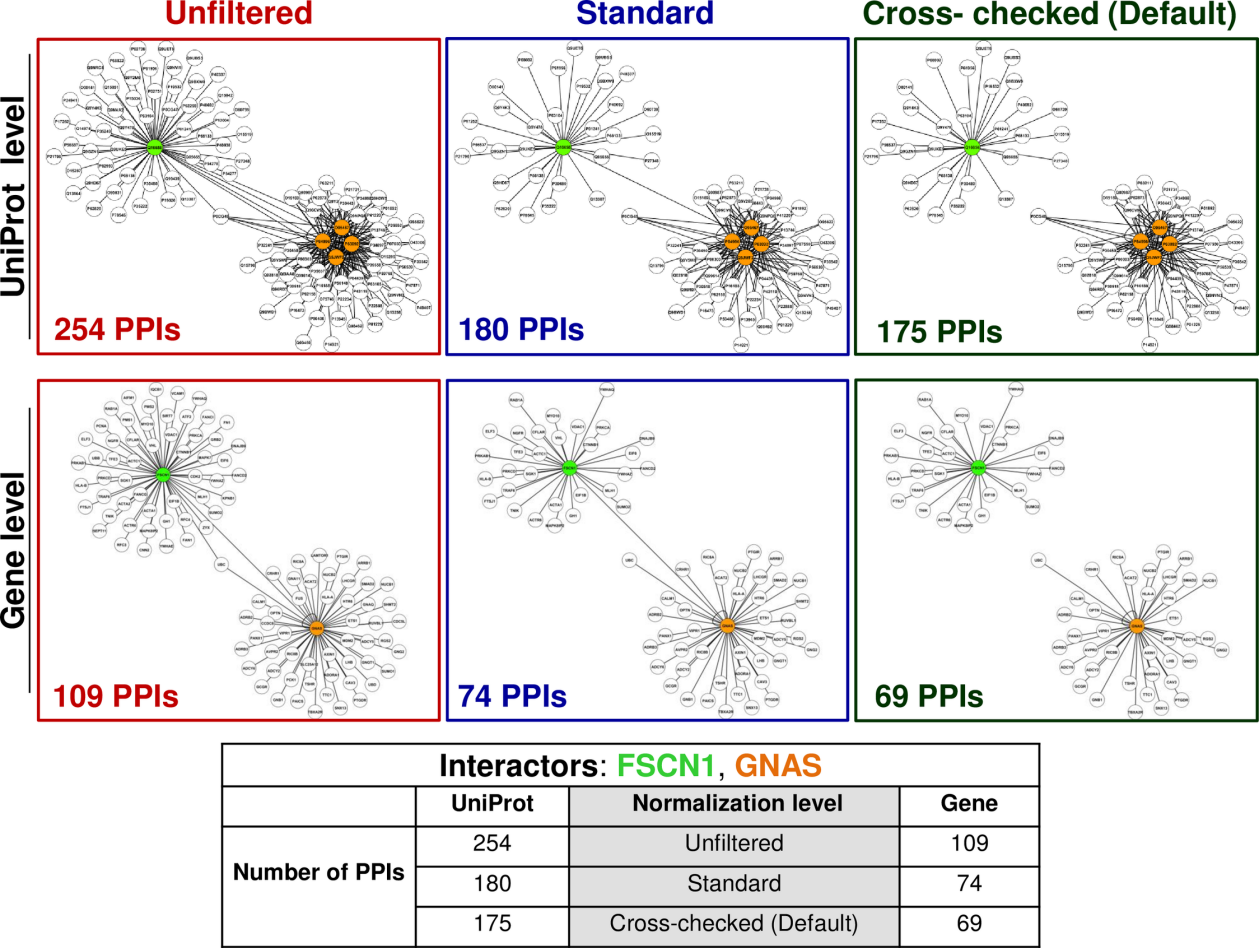
The Fascin actin-bundling protein (FSCN1) and the GNAS complex locus protein (GNAS) interaction network in PICKLE 2.0 at the UniProt and gene levels and all three filtering modes (unfiltered, standard, cross-checked (default)). There is a clear decrease in the number of PPIs at both levels moving from the unfiltered to the cross-checked (default) filtering mode.

When examining the BioGRID- and IntAct- provided experimental evidence in combination, it is observed that both evidence sets are derived from the same publication (PubMed: 17353931). Clearly, the two evidences are not independent and their apparent differences that lead to conflicting standard quality designations are due to distinct curation and annotation protocols by the two PPI sources. Through the evidence cross-examination between BioGRID and IntAct, the cross-checked quality of the IntAct-reported evidence is down-graded to third-class, and the interaction between O95467 and P63092 is assigned to the third cross-checked confidence threshold that leads it out of the cross-checked PICKLE 2.0 network. By examining all these curation overlaps, the cross-checked (default) PICKLE 2.0 network is reduced by about 4,000 interactions compared to the standard interactome at both levels of genetic reference (**S1** and **S2 Files**). Specifically for the discussed PPI example, the network of the first neighbors of the involved proteins in the unfiltered PICKLE 2.0 at the UniProt level comprises 254 PPIs (**Fig 5**), including the 23 BioGRID-reported interactions of the *FSCN1* Entrez Gene ID being accounted four times for the respective four UniProt_IDs to which the *FSCN1* Entrez Gene ID ontologically corresponds. In the standard UniProt-normalized PICKLE interactome, the respective network is now reduced to 180 PPIs, with only one interaction connecting the Fascin actin-bundling UniProt_ID with one UniProt_ID of the GNAS complex locus protein. Finally, in the cross-checked(default) PICKLE, the network gets reduced even further down to 175 PPIs and holds no interaction between the two proteins (**Fig 5**). Clearly, the corresponding networks at the gene level are of substantially smaller size, i.e. 109, 74 and 69 PPIs, respectively, in the unfiltered, standard and cross-checked (default) (**Fig 5**), as there is no “inflation” due to the normalization of the BioGRID reported PPIs for the *FSCN1* Entrez Gene ID at the UniProt level.

## Supporting information

S1 Table Entity types and their attributes stored in PICKLE 2.0 from primary biological databases. (DOCX)

S2 Table The sets of evidence attributes recorded from each source PPI database. (DOCX)

S3 Table The ruleset for the standard filtering of the primary PPI datasets. (DOCX)

S4 Table Comparison of the RHCPs used in PICKLE 2.0 (release1) and PICKLE 1.0. (A) The common RHCP UniProt IDs in the RHCP used in PICKLE 2.0 (release 1) and PICKLE 1.0; (B) The differences in the RHCP used in PICKLE 2.0 (release 1) compared to PICKLE 1.0 (provided as a separate Excel file with two respective sheets).
(XLSX)

S5 Table The number of interactions (degree) of the RHCP UniProt_IDs included in the PICKLE 2.0 (release 1) and PICKLE 1.0 interactomes. (XLSX)

S6 Table The correspondences between the UniProt_IDs and Entrez Gene IDs of the default PICKLE 2.0 (release 1) network at the two normalization levels. (A) The UniProt IDs in PICKLE 2.0 with no associated Entrez Gene ID; (B) The UniProt IDs in PICKLE 2.0 associated with multiple Entrez Gene IDs; (C) The Entrez Gene IDs in PICKLE 2.0 associated with multiple UniProt IDs; (provided as a separate Excel file with three respective sheets).
(XLSX)

S1 File Assessing the way of expansion of the RHCP PPI network: PICKLE 2.0 (release 1) vs. PICKLE 1.0. (DOCX)

S2 File Comparison of the PICKLE 2.0 (release 1) network between the UniProt and gene levels. (DOCX)
