# Supplementary material for: PICKLE 2.0: A human protein-protein interaction meta-database employing data integration via genetic information ontology
Source: PLoS One. 2017 Oct 12;12(10):e0186039. doi: 10.1371/journal.pone.0186039 (PMC5638325; doi:10.1371/journal.pone.0186039)
Supplement: S1 Table — (DOCX) [file pone.0186039.s001.docx]

S1 Table. Entity types and their attributes stored in PICKLE 2.0 from primary biological databases

| **UniProt Entity** | **Nucleotide Sequence (mRNA) Entity defined by RefSeq nucleotide ID** | **Nucleotide Sequence (mRNA) Entity defined by EMBL nucleotide ID** | **Gene Entity defined by Entrez Gene ID** | **Gene Entity defined by Ensembl gene ID** |
| --- | --- | --- | --- | --- |
| -Primary accession ^§^  -Secondary accession ^§^  -Entry name ^§^  -Protein name ^§^  -Review status ^§^  -Evidence level  -Sequence length  -Sequence status  -TaxID  -Creation date  -Last modification date  -Isoform IDs ^§^  -GO codes  -Keywords  -All cross-references | -RefSeq nucleotide ID (*UniProt*) ^§ [1]^  -Secondary nucleotide IDs ^§^  -Version  -Nucleotide GI ^§^  -Name ^§^  -Molecule type  -Topology  -Sequence length  -TaxID  -Creation date  -Last modification date  -RefSeq protein accession ^§^  -RefSeq protein GI ^§^ | -EMBL nucleotide ID (*UniProt*) ^§ [1]^  -Version  -Name ^§^  -Molecule type  -Topology  -Sequence length  -TaxID  -Creation date  -Last modification date | -Entrez Gene ID (*UniProt*) ^§ [1]^  -Deprecated Gene ID ^§^  -Primary symbol ^§^  -Deprecated Symbol ^§^  -Synonyms ^§^  -Name ^§^  -Chromosome  -Locus  -Gene type  -Source genome  -Source origin  -TaxID  -Creation date  -Last modification date | -Ensembl gene ID (*UniProt*) ^§ [1]^  -Primary symbol ^§^  -Name ^§^  -Chromosome  -TaxID  -Last modification date |

**^[1]^** UniProt, GenBank Gene, GenBank Nucleotide, ENA and Ensembl are the biological sources for UniProt entity, Gene Entity defined by Entrez Gene ID, nucleotide sequence (mRNA) entity defined by RefSeq nucleotide ID, nucleotide sequence (mRNA) entity defined by EMBL nucleotide ID and Gene Entity defined by Ensembl gene ID, respectively, unless otherwise specified in parenthesis.

**^§^** Included in the OWL ontology; in the latter, the UniProt Entity comprises two subclasses: a) the SwissProt (reviewed) and b) the TrEMBL (unreviewed) subclass. The RHCP ontological network exclusively comprises SwissProt UniProt entries.
