# Supplementary material for: PICKLE 2.0: A human protein-protein interaction meta-database employing data integration via genetic information ontology
Source: PLoS One. 2017 Oct 12;12(10):e0186039. doi: 10.1371/journal.pone.0186039 (PMC5638325; doi:10.1371/journal.pone.0186039)
Supplement: S2 Table — (DOCX) [file pone.0186039.s002.docx]

S2 Table. The sets of evidence attributes recorded from each source PPI database

| **HPRD** | **BioGRID** | **IntAct** | **MINT** | **DIP** |
| --- | --- | --- | --- | --- |
| -Detection Method | -Interaction ID  -Interaction Type  -Detection Method  -Experimental System  -Throughput | -Interaction ID  -Interaction Type  -Detection Method  -Expansion Method | -Interaction ID  -Interaction Type  -Detection Method  -Expansion Method | -Interaction ID  -Interaction Type  -Detection Method  -Throughput |
