# Supplementary material for: PICKLE 2.0: A human protein-protein interaction meta-database employing data integration via genetic information ontology
Source: PLoS One. 2017 Oct 12;12(10):e0186039. doi: 10.1371/journal.pone.0186039 (PMC5638325; doi:10.1371/journal.pone.0186039)
Supplement: S3 Table — (DOCX) [file pone.0186039.s003.docx]

**S3 Table. The ruleset for the standard filtering of the primary PPI datasets**.

The table indicates the evidence sets that are considered in the various quality classes regarding the probability of suggesting a direct PPI, based on the evidence attributes reported by each source database (shown in **S2 Table**).

| **Quality** | **Experimental Evidence Sets stored in PICKLE for cross-checking purposes only;**  **not included in the unfiltered PICKLE PPI network** | | | | | **Database** |
| --- | --- | --- | --- | --- | --- | --- |
|  | **Interactor Type** | **Interaction Type** | **Experimental System (BioGRID) or**  **Detection Method (HPRD, MIntAct, DIP)** | **Throughput** | **Expansion**  **Method** |  |
| Quinary-class | Any applicable | Any applicable | Any of the following:   - - - - Protein-RNA       - Affinity Capture RNA | Any |  | BioGRID |
| Quinary-class | At least one interactor is not protein or peptide | Any applicable | Any applicable |  | Any | MIntAct |

| **Quality** | **Experimental Evidence Set included in the unfiltered PICKLE PPI network** | | | | **Database(s)** |
| --- | --- | --- | --- | --- | --- |
|  | **Interaction Type** | **Experimental System (BioGRID) or**  **Detection Method (HPRD, MIntAct, DIP)** | **Throughput** | **Expansion**  **Method** |  |
| First-class | 1. Any of the following:  - direct interaction - adp ribosylation reaction - acetylation reaction - cleavage reaction - covalent binding - deacetylation reaction - demethylation reaction - dephosphorylation reaction - deubiquitination reaction - enzymatic reaction - gtpase reaction - hydroxylation reaction - methylation reaction - neddylation reaction - palmitoylation reaction - phosphorylation reaction - phosphotransfer reaction - protein cleavage - ubiquitination reaction | Any | Any  (if applicable) | Any  (if applicable) | BioGRID, MInAct, DIP |
| First-class | 1. Any other interaction type than those mentioned in criterion 1 of first class above (among the evidence sets included in the unfiltered PICKLE PPI network). | Any of the following:   - PCA (BioGRID) - protein-peptide (BioGRID) - FRET (BioGRID) - two-hybrid (BioGRID) - reconstituted Complex (BioGRID) - biochemical activity (BioGRID) - antibody array - beta galactosidase complementation - bimolecular fluorescence complementation - bioluminescence resonance energy transfer - competition binding - deacetylase assay - dihydrofolate reductase reconstruction - enzymatic study - enzyme linked immunosorbent assay - equilibrium dialysis - far western blotting - filter binding - fluorescence polarization spectroscopy - fluorescence recovery after photobleaching - fluorescent resonance energy transfer - gal4 vp16 complementation - gtpase assay - isothermal titration calorimetry - lex-a dimerization assay - mammalian protein protein interaction trap - methyltransferase assay - methyltransferase radiometric assay - peptide array - phage display - protein array - protein kinase assay - reverse ras recruitment system - reverse two hybrid - split luciferase complementation - split renilla luciferase complementation - three-hybrid - tox-r dimerization assay - transcriptional complementation assay - two-hybrid (the only applicable to HPRD; should be one of the supporting detection methods) - two-hybrid array - ubiquitin reconstruction - x-ray crystallography - yeast display | Any  (if applicable) | Any  (if applicable) | BioGRID, MInAct, DIP |
| Second-class | Any other than interaction type than those mentioned in criterion 1 of first-class above. | Any other than experimental system (BioGRID) or detection method (MIntAct, DIP) than those mentioned in criterion 2 of first-class above; any set of supporting detection methods not including ‘two-hybrid’ (HPRD). | Low or  both (Low & High)  (when applicable) | No  (when applicable) | BioGRID, HPRD, MIntAct, DIP |
| Third-class | Any other than interaction type than those mentioned in criterion 1 of first-class above. | Any other than experimental system (BioGRID) or detection method (MIntAct, DIP) than those mentioned in criterion 2 of first-class above; any set of supporting detection methods not including ‘two-hybrid’ (HPRD). | High (only)  (when applicable) | Yes  (when applicable) | BioGRID, HPRD, MIntAct, DIP |
