# Supplementary material for: PICKLE 2.0: A human protein-protein interaction meta-database employing data integration via genetic information ontology
Source: PLoS One. 2017 Oct 12;12(10):e0186039. doi: 10.1371/journal.pone.0186039 (PMC5638325; doi:10.1371/journal.pone.0186039)
Supplement: S1 File — (DOCX) [file pone.0186039.s007.docx]

# S1 File

# Assessing the way of expansion of the RHCP PPI network: PICKLE 2.0 (release 1) vs. PICKLE 1.0

## The RHCP PPI network in PICKLE 2.0 (release 1)

The PICKLE 2.0 PPI network (release 1) has been reconstructed based on an instantiated genetic information ontology network formed from the information provided by the UniProtKB RHCP release 2015_01, combined with information from GenBank (Release 211), ENA (release 122) and Ensembl (release 83); the ontological network is available in OWL format at the PICKLE website.

The PICKLE PPI network integrates:

(a) the HPRD binary PPI dataset (release 9), using the file ‘HPRD_ID_MAPPINGS’ to correspond each hprd ID to one nucleotide sequence (mRNA) ID (either RefSeq ID or EMBL ID),

(b) the IntAct- and (c) the MINT- designated datasets of the MIntAct PSI-MI tab-delimited file (release 2015-02-23), after removing the entries for which at least one interactor is non-human, i.e. the taxon ID (TaxID) is not 9606,

(c) the BioGRID PSI-MI tab-delimited file (release 3.2.120) for human using information from both the mitab and tab2 versions, and

(d) the DIP PSI-MI tab delimited file (release 20150101) for human.

Τhe statistics of the unfiltered, standard and cross-checked (default) PICKLE 2.0 (release 1) networks at the UniProt level are shown in **Table A** in **S1 File**. The default PICKLE 2.0 (release 1) interactome covers 14134 (~70%) out of 20193 RHCP UniProt IDs with 120,882 PPIs supported by 35752 references, mainly, PubMed-cited publications (**Table A** in **S1 File**). The main supplier of the default PICKLE 2.0 network is BioGRID contributing 91% of the UniProt IDs and ~75% (43.5% as the unique source) of the PPIs (**Table B** in **S1 File**). The standard PICKLE 2.0 formed from the integration of the source PPI datasets supported by at least one “first-class” or “second-class” evidence set, excluding the cross-evaluation between the data sources, covers 59 more RHCP UniProt IDs and includes 3989 additional interactions compared to the default. Most of these additional PPIs are supplied from BioGRID and Intact, which are the two mainly cross-checked datasets, and a moderate number of PPIs (385) from HPRD. Furthermore, the unfiltered PICKLE 2.0 network covers 1532 RHCP UniProt IDs than the standard PICKLE network and includes 66776 additional interactions (~75% and ~9% supplied uniquely from BioGRID and MIntAct, respectively), which are supported only by “tertiary-class” evidence sets (see **Tables A**-**D** in **S1 File**). The number of common references between the primary datasets remains very small, e.g. only 556 (1.5% of the total) common references were identified between the three largest datasets (BioGRID, IntAct and HPRD) in the default network (**Table B** in **S1 File**). This observation further supports the need for multiple source PPI database integration for the reconstruction of a comprehensive human protein interactome. Markedly, 81% of the references supporting the default PICKLE 2.0 PPIs are supplied uniquely from BioGRID and/or HPRD.

Table A. The statistics of PICKLE 2.0 (release 1) at the UniProt level and the three filtering modes (unfiltered, standard and cross-checked (default)). The RHCP size at the UniProt level is also shown. The statistics include self-loops.

|  | **UniProt level** | | |
| --- | --- | --- | --- |
|  | **UniProt IDs** | **PPIs** | **References** |
| **RHCP size** | 20193 | - | - |
| **Unfiltered** | 15725 | 191647 | 35930 |
| **Standard** | 14193 | 124871 | 35774 |
| **Cross-checked (Default)** | 14134 | 120882 | 35752 |

Table B. Source of the data in the cross-checked (default) PICKLE 2.0 (release 1) PPI network at the UniProt level.


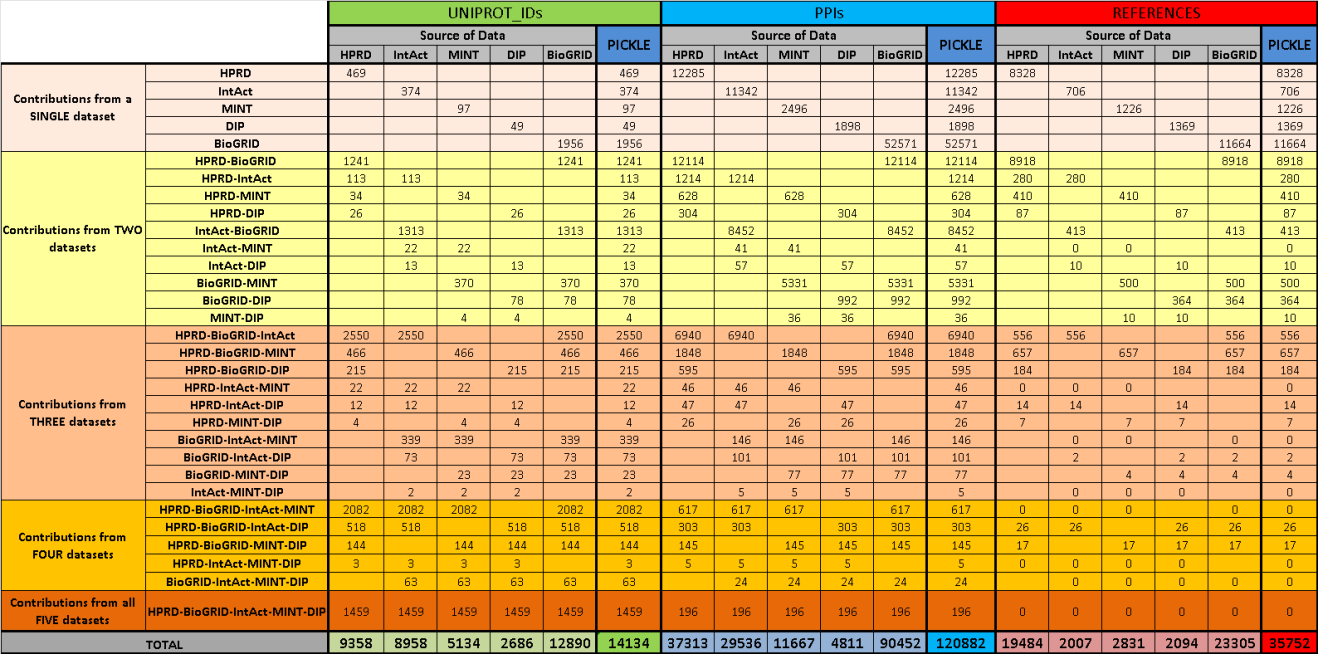


Table C. Source of the data in the standard PICKLE 2.0 (release 1) PPI network at the UniProt level.


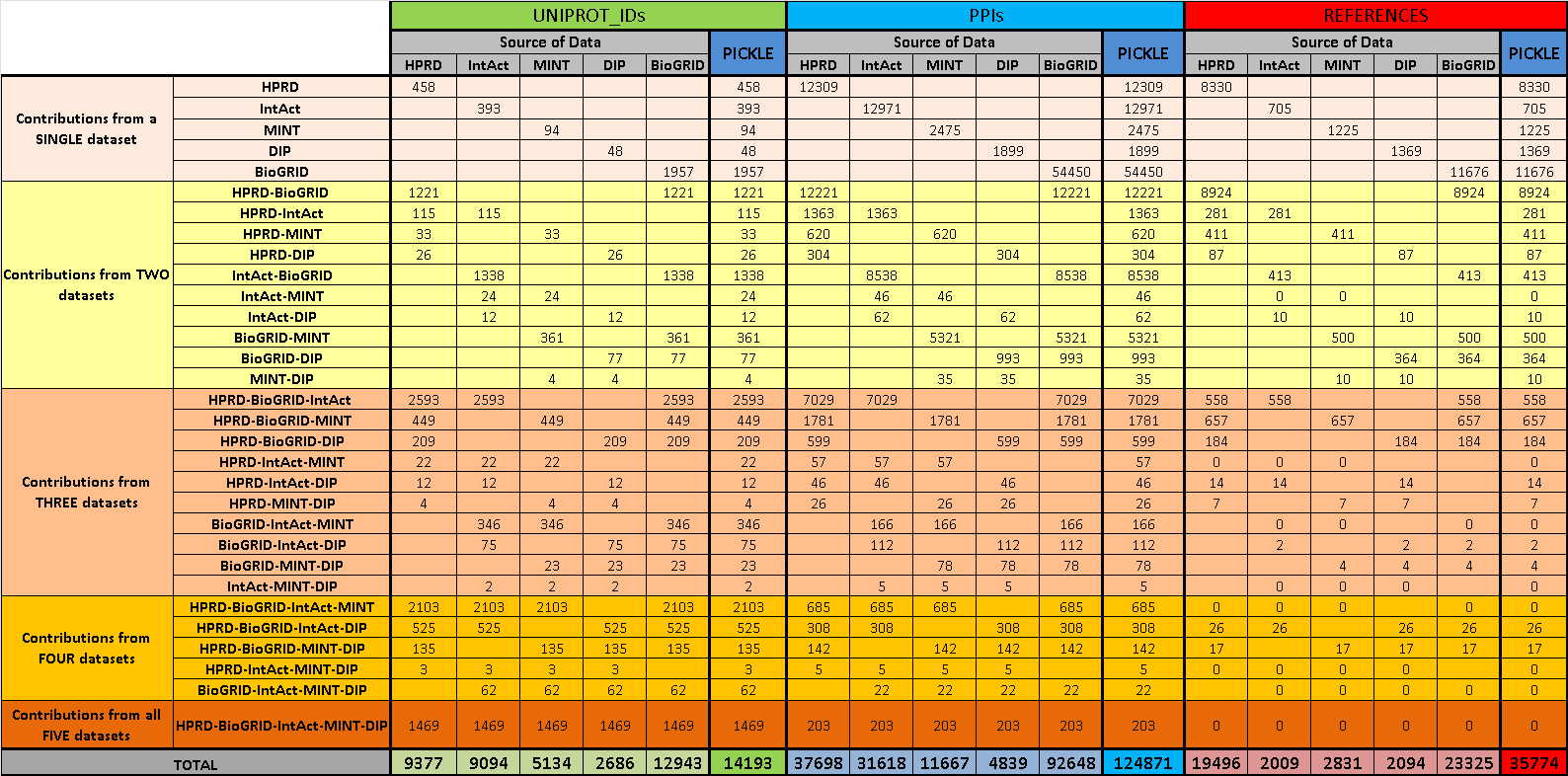


Table D. Source of the data in the unfiltered PICKLE 2.0 (release 1) PPI network at the UniProt level.


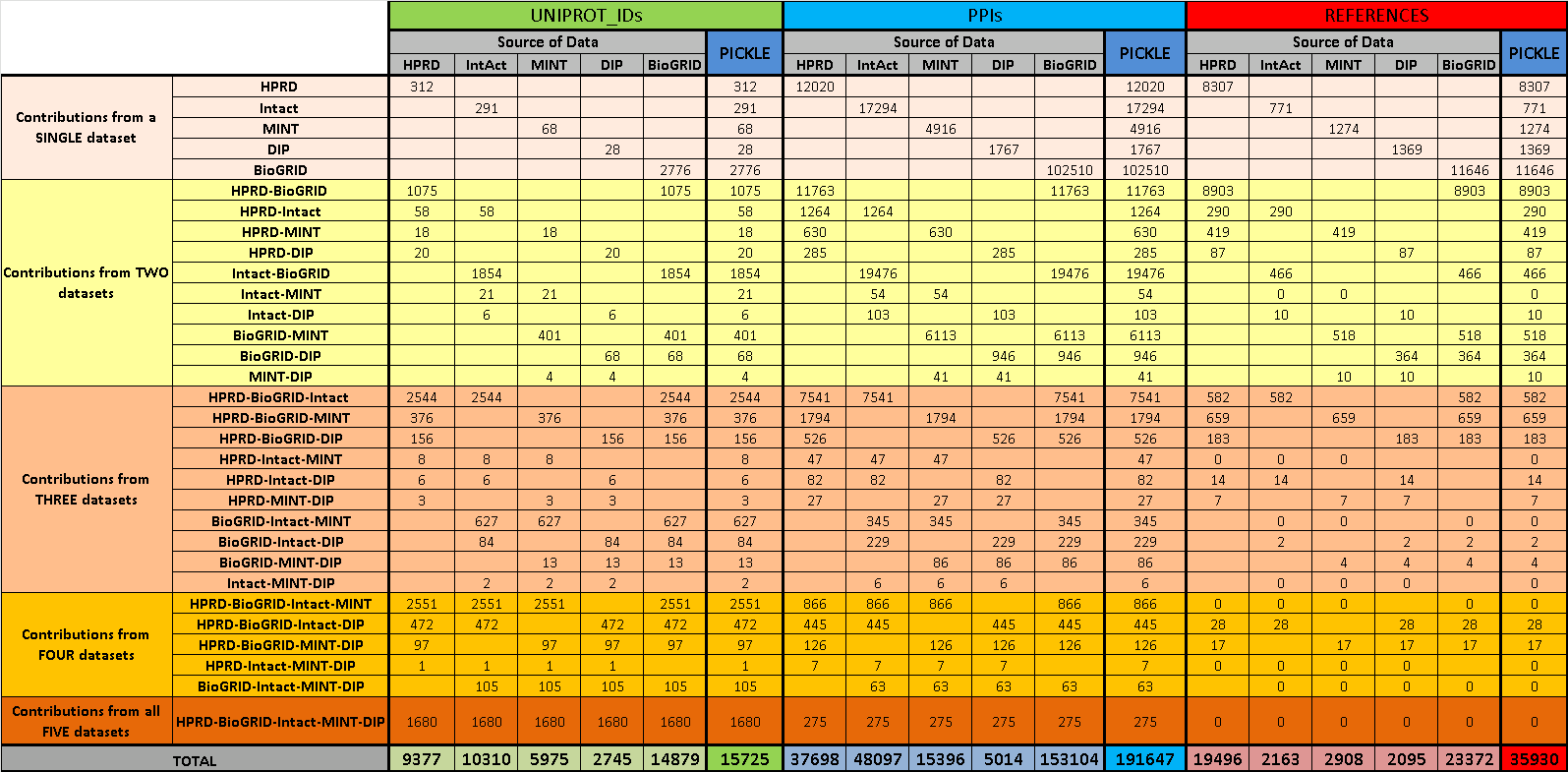


## PICKLE 2.0 (release 1) vs. PICKLE 1.0

**Table A** in **S1 File** shows that, on the basis of an almost identical RHCP (see full comparison of the RHCP used in PICKLE 2.0 (release 1) compared to PICKLE 1.0 in **S4 Table** – provided in a separate Excel file), the RHCP PPI network in PICKLE 2.0 (release 1) covers 2307 more UniProt IDs, corresponding to 11.4% of the RHCP, and includes 44917 additional protein interactions than in 2013, i.e. an increase of 59.1% with respect to PICKLE 1.0 (see also **Table F** in **S1 File**). This increase trend supports our previous statement while analyzing PICKLE 1.0, that new experiments and incorporation of new references are expected to reveal additional interactions concerning mostly the proteins already participating in the interactome, while most of the newly added UniProt IDs will have fewer than four interactions [10]. Indeed, comparison of the degree distribution of PICKLE 2.0 (release 1) vs. PICKLE 1.0 (see **S5 Table**, showing the degree of each interactor in PICKLE 2.0 (release 1) cross-checked (default) and in PICKLE 1.0, provided in a separate Excel file), indicates that this is the case for most (83%) of the 2427 newly added UniProt IDs in the network of PICKLE 2.0; it is noted that 2411 of these UniProt IDs are common in the RHCPs used for the construction of PICKLE 1.0 and PICKLE 2.0. Further comparison of the power-law curves characterizing PICKLE 2.0 and PICKLE 1.0 (**Figs A** and **B** in **S1 File**) indicates that most (63%) of the newly added interactions increase the number of UniProt IDs with degree larger than 10 and smaller than or equal to 100. Moreover, in PICKLE 2.0, there is an increase in the number of UniProt IDs with more than 300 PPIs (i.e. 37 compared to 16 in PICKLE 1.0, **Fig B** in **S1 File**, **S5 Table**). The larger number of hubs in PICKLE 2.0 is in agreement with the network analysis results (**Table E** in **S1 File**), mainly with the increased clustering coefficient, average number of neighbors, network centralization and number of shortest paths.

The observed expansion of the experimentally supported human protein interactome in PICKLE 2.0 compared to PICKLE 1.0 is mainly due to the meanwhile increase in the PPIs stored in IntAct and BioGRID (**Table F** in **S1 File**). Namely, the RHCP-related data of these source databases have, respectively, increased by 34.4% and 39.1% in UniProt IDs, 52.1% and 112.1% in direct PPIs and 25.6% and 68.7% in supporting references. DIP, the smallest source database, has appreciably increased its size by 49.6% in UniProt IDs, 84.4% in PPIs and 77.5% in references.

**
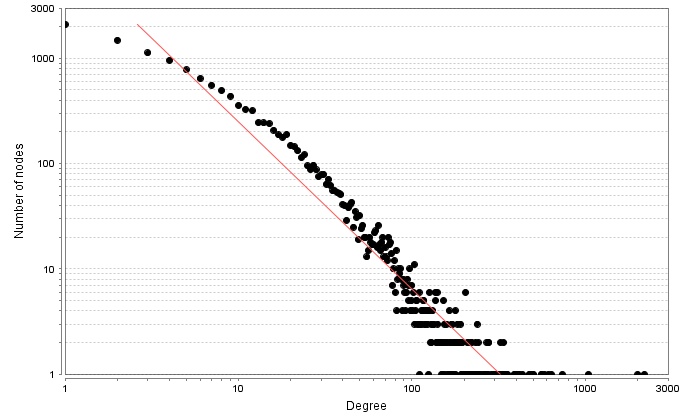
**

number of UniProt IDs with x PPIs = 9519 x^-1.585^

R^2^ (on logarithmized values) = 89.3%

**number of PPIs (UniProt ID degree)**

**number of UniProt IDs with *x* PPIs**

P0CG48

P05067

P61956

**Fig A. The degree (number of interactions) distribution in the default PICKLE 2.0 (release 1) at the UniProt level.** The red line indicates the power-law fit (logarithmic scale); the related equation and R^2^ correlation are also shown. Curve and fit was produced and uploaded from the network analysis tool of Cytoscape (2.8.2).


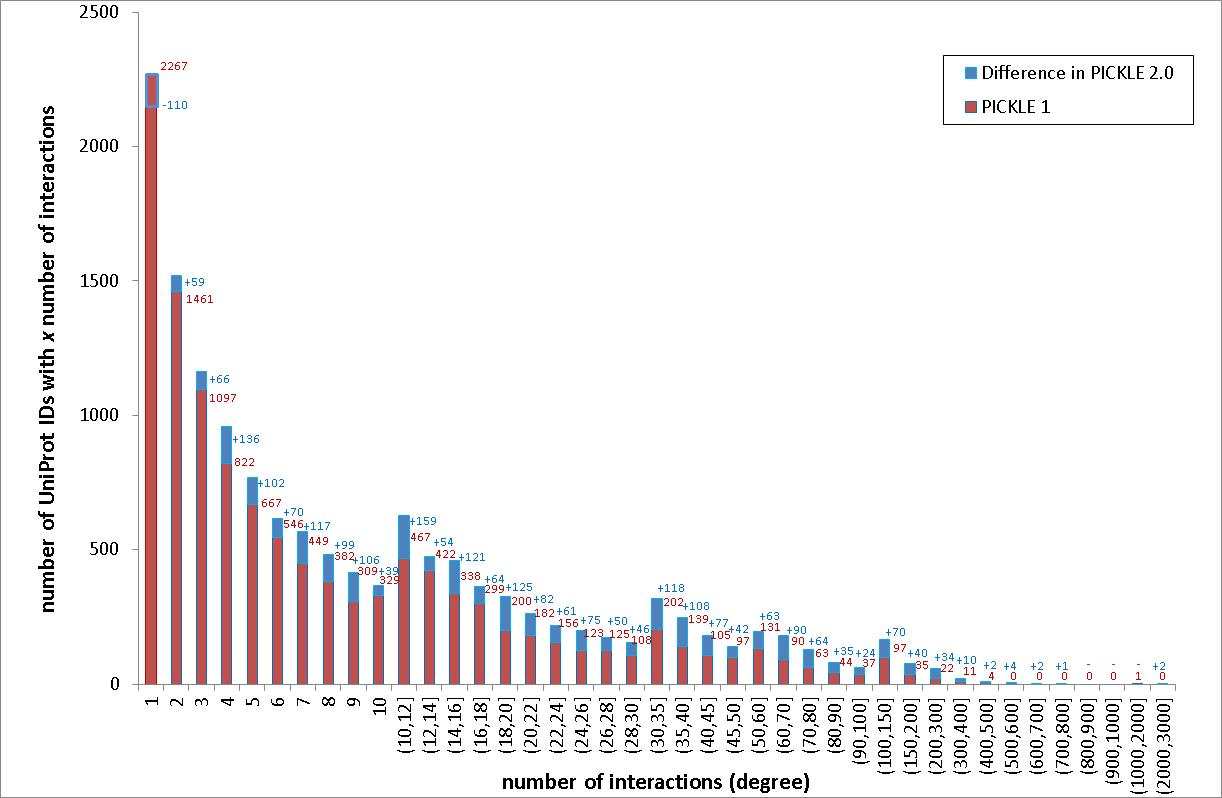


**Fig B. The degree (number of interactions) distribution in cross-checked (default) PICKLE 2.0 (release 1) vs. PICKLE 1.0.**

Table E: The characteristics of the PICKLE 2.0 (release 1) default network (at the UniProt level) compared to PICKLE 1.0.

| **Network Characteristic ^(1)^** | **PICKLE 1.0 Value ^(2)^** | **PICKLE 2.0 Value ^(2)^** |
| --- | --- | --- |
| Number of Nodes | 11827 | 14134 |
| Isolated Nodes (homodimers) | 114 | 68 |
| Connected components | 174 (i.e: 1 cluster of 11577 nodes, 114 homodimers, 46 heterodimers, 13 isolated of 3 or 4 nodes). | 95 (i.e: 1 cluster of 14010 nodes, 68 homodimers, 22 heterodimers, 4 three-node isolated components). |
| Number of self-loops | 2715 (i.e.: 2601 nodes having interactions with other proteins as well, and 114 isolated homodimers). | 3515 (i.e.: 3447 nodes having interactions with other proteins too, and 68 isolated homodimers). |
| Network radius | 1 | 1 |
| Network diameter | 12 | 10 |
| Characteristic Path Length | 3.691 | 3.350 |
| Average Number of Neighbors | 12.387 | 16.608 |
| Shortest Paths | 95% | 98% |
| Clustering Coefficient | 0.127 | 0.134 |
| Network Density | 0.001 | 0.001 |
| Network Centralization | 0.093 | 0.153 |
| Network Heterogeneity | 2.193 | 2.567 |

^(1)^ Detailed Description for every network characteristic can be found in

*http://med.bioinf.mpi-inf.mpg.de/netanalyzer/help/2.6.1/index.html*

^(2)^ Determined using the network analysis tool of Cytoscape (2.8.2)

Table F: The rate of expansion of the default PICKLE network (UniProt level) and the contributing primary PPI datasets from PICKLE 1.0 to PICKLE 2.0 (release 1).

|  | **UniProt IDs** | | | **PPIs** | | | **References** | | |
| --- | --- | --- | --- | --- | --- | --- | --- | --- | --- |
|  | **[PICKLE 1.0]** | **[PICKLE 2.0]** | **difference^[1]^** | **[PICKLE 1.0]** | **[PICKLE 2.0]** | **difference** | **[PICKLE 1.0]** | **[PICKLE 2.0]** | **difference** |
| **RHCP** | 20242 | 20193 | - 0.24% ^[2]^ |  |  |  |  |  |  |
| **HPRD** | 9303 | 9358 | +0.6% | 37152 | 37313 | +0.4% | 19267 | 19484 | +1.1% |
| **IntAct** | 6666 | 8958 | +34.4% | 19425 | 29536 | +52.1% | 1598 | 2007 | +25.6% |
| **MINT** | 6102 | 5134 | -15.9% | 16147 | 11667 | -27.7% | 2398 | 2831 | +18.1% |
| **DIP** | 1795 | 2686 | +49.6% | 2609 | 4811 | +84.4% | 1180 | 2094 | +77.5% |
| **BioGRID** | 9265 | 12890 | +39.1% | 42647 | 90452 | +112.1% | 13818 | 23305 | +68.7% |
| **PICKLE** | **11827** | **14134** | **+19.5%** | **75965** | **120882** | **+59.1%** | **26689** | **35752** | **+34.0%** |

**^[1]^** The % difference is calculated with respect to the size of the corresponding entity in PICKLE 1.0.

**^[2]^** The differences between the UniProtKB-defined RHCP used in PICKLE 1.0 and PICKLE 2.0 (release 1) are shown in **S4 Table**.
