# Supplementary material for: PICKLE 2.0: A human protein-protein interaction meta-database employing data integration via genetic information ontology
Source: PLoS One. 2017 Oct 12;12(10):e0186039. doi: 10.1371/journal.pone.0186039 (PMC5638325; doi:10.1371/journal.pone.0186039)

# S2 File

# Comparison of the PICKLE 2.0 (release 1) network between the UniProt and gene levels

**Table A** in **S2 File** shows the statistics of the PICKLE 2.0 (release 1) network at the gene level and the three different filtering modes. **Table B** in **S2 File** indicates the contributions of the primary PPI datasets to the cross-checked (default) PICKLE 2.0 (release 1). Comparison with the corresponding data for the UniProt level (**Tables A** and **B** in **S1 File**) indicates that there is no substantial overall difference in the size of the human protein interactome between the two levels of genetic reference; noting that it is by ~1550 PPIs larger at the gene than at the UniProt level in the standard filtering and cross-checking modes. More specifically, the default PICKLE 2.0 (release 1) network comprises 120,882 interactions between 14134 UniProt IDs and 122,429 interactions between 14107 Entrez Gene IDs. This similarity is mainly due to the fact that 117,640 (97.3% at the UniProt level, 96.1% at the gene level) PPIs have a single “sister” interaction at the antipodal level, because 13747 of the 14134 UniProt IDs have a single associated Entrez Gene ID among the 14107, and vice versa.

However, in certain regions, the PICKLE network instances at the UniProt and gene level have substantial differences that need to be taken into consideration, as the two networks are not isomorphic and cannot be used interchangeably. These differences are:

1. Certain PPIs (307, i.e. 0.254% of the total) of the UniProt-normalized human protein interactome have no “sister” interaction in the gene-normalized network, because at least one of their interactors belongs to the 1326 RHCP UniProt entries with no associated Entrez Gene ID in the PICKLE 2.0 ontological network, according to the associations made by UniProtKB in its utilized release (see note in **Table A** in **S2 File**). The default PICKLE PPI network includes 106 of these UniProt entries, shown in **S6 Table** (Part A) (provided in a separate Excel File) along with the number of their interactions.
2. The default PICKLE 2.0 (release 1) PPI network includes 173 UniProt IDs associated with multiple (up to 14) Entrez Gene IDs and 84 Entrez Gene IDs with multiple (up to 7) UniProt IDs, shown in **S6 Table** (Parts B and C), respectively, (provided in a separate Excel File) along with the number of their interactions. It should be noted that in the PICKLE RHCP ontological network, we also infer UniProt ID – Entrez Gene ID associations that are not directly made by UniProtKB in its utilized release, through the associations UniProt ID – Ensembl ID (uploaded by UniProtKB), or UniProt ID – EMBL nucleotide sequence (mRNA) ID (uploaded by UniProtKB) and Ensembl ID - Entrez Gene ID (uploaded by GenBank). These inferred associations are also used in the normalization of the integrated hybrid PICKLE PPI network to the UniProt or gene levels.

Table A. The statistics of PICKLE 2.0 (release 1) at the gene level and the three filtering modes (unfiltered, standard and cross-checked (default)). The RHCP size at the gene level is also shown. The statistics include self-loops.

|  | **Gene level** | | |
| --- | --- | --- | --- |
|  | **Entrez Gene IDs** | **PPIs** | **References** |
| **RHCP size** | 19042 **^[1]^** | - | - |
| **Unfiltered** | 15652 | 193947 | 35882 |
| **Standard** | 14167 | 126469 | 35727 |
| **Cross-checked (Default)** | 14107 | 122429 | 35705 |

^[1]^ The number of Entrez Gene IDs assigned by UniProtKB to the RHCP UniProt entries in the utilized UniProtKB release. It should be noted that in the PICKLE 2.0 (release 1) genetic information ontology network, there exist 1326 RHCP UniProt IDs with no associated Entrez Gene ID, according to the associations provided by UniProtKB in its utilized release. Specifically, 966 RHCP UniProt IDs are not associated to any gene identifier (Entrez Gene ID or Ensembl ID). For additional 379 RHCP UniProt IDs, UniProtKB provides solely association to an Ensembl ID; this association enabled us to infer association to an Entrez Gene ID for 19 of these 379 UniProt IDs based on the GenBank exported information about Ensembl ID – Entrez Gene ID associations. 360 RHCP UniProt IDs remained with no associated Entrez Gene ID.

Table B. Source of the data in the cross-checked (default) PICKLE 2.0 (release 1) PPI network at the gene level


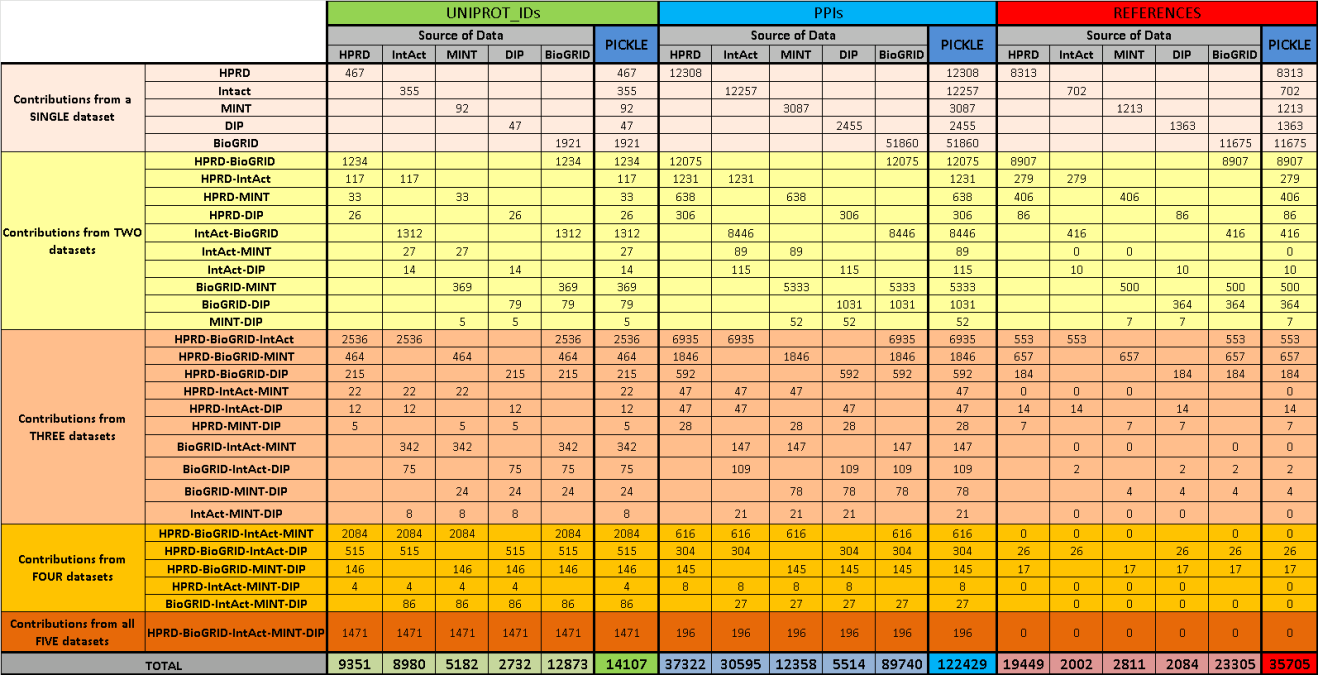

Supplement: S2 File — (DOCX) [file pone.0186039.s008.docx]
